# Soluble expression, purification, and characterization of active recombinant human tissue plasminogen activator by auto-induction in *E. coli*

**DOI:** 10.1186/s12896-015-0127-y

**Published:** 2015-03-01

**Authors:** Xiaobin Long, Yeran Gou, Miao Luo, Shaocheng Zhang, Hongpeng Zhang, Lei Bai, Shuang Wu, Quan He, Ke Chen, Ailong Huang, Jianzhong Zhou, Deqiang Wang

**Affiliations:** Department of Cardiology, The First Affiliated Hospital of Chongqing Medical University, Chongqing, 400016 China; Key Laboratory of Molecular Biology on Infectious Disease (Ministry of Education), The Second Affiliated Hospital, Chongqing Medical University, Chongqing, 400016 China; Key Laboratory of Clinical Laboratory Diagnostics (Ministry of Education), College of Laboratory Medicine, Chongqing Medical University, Chongqing, 400016 PR China; Department of Clinical Laboratory, Yubei District People’s Hospital, Chongqing, 401120 PR China

**Keywords:** tPA, Recombinant protein, Soluble expression, Autoinduction, Thrombus

## Abstract

**Background:**

Human tissue plasminogen activator (tPA) belongs to the serine protease family. It converts plasminogen into plasmin and is used clinically to treat thrombosis. Human tPA is composed of 527 amino acids residues and contains 17 disulfide bonds. *Escherichia coli* has been used only rarely for the efficient production of recombinant tPA. However, the functional expression of full-length tPA that contains multiple disulfide bonds on an industrial scale remains challenging. Here, we describe the soluble expression and characterization of full-length tPA by auto-induction in *E. coli*.

**Results:**

We achieved optimal levels of gene expression, minimized negative effects related to the production of heterologous proteins, and optimized cytoplasmic yields. Three different *E. coli* strains, BL21 (DE3), Rosetta, and Origami 2, could express tPA using an auto-induction mechanism. In addition, similar yields of recombinant protein were produced at temperatures of 33, 35, and 37°C. The *E. coli* strain origami 2 could increase disulfide bond formation in cytoplasmic tPA and produce purified soluble recombinant protein (~0.9 mg/l medium). The full-length tPA was monomeric in solution, and fibrin plate assays confirmed that the recombinant tPA displayed serine protease activity.

**Conclusions:**

This is the first report that describes the heterologous expression of correctly folded active full-length tPA. This could provide valuable information for using prokaryotic auto-induction expression systems to produce tPA at industrial and pharmaceutical levels without *in vitro* refolding during the production step.

## Background

Human tissue plasminogen activator (tPA), a serine protease that selectively cleaves plasminogen, is found in the fibrinolytic system of blood vessel endothelial cells [1]. The human protein comprises 527 amino acids residues, including 35 cysteine residues that participate in the formation of 17 disulfide bonds [1]. tPA contains five distinct structural domains: an N-terminal finger domain (F domain, residues 4–50), the epidermal growth factor-like domain (E domain, residues 50–87), two kringle domains (K1 domain, residues 87–176; and K2 domain, residues 176–256), and a serine protease catalytic domain (P domain, residues 276–527). Its binding to fibrin and the subsequent modulation of protease activity are regulated primarily by the F and K2 domains, respectively. tPA converts plasminogen into plasmin, and has been used clinically to treat thrombosis [2].

However, the functional preparation of tPA containing multiple disulfide bonds remains the bottleneck for its production on an industrial scale. The expression and purification of human tPA from eukaryotic and prokaryotic sources, such as human uterus or Chinese hamster ovary cells, has been described in several reports [3-5]. In contrast, tPA that is purified from mammalian expression systems is expensive, and the resulting glycosylated protein is cleared rapidly from the blood [6]. Consequently, several recombinant hosts, such as *Saccharomyces cerevisiae* and insect systems, have been used for the industrial preparation of tPA. However, these have also been associated with several problems including hyperglycosylation, poor export, and improper folding [7-10].

Due to its low cost and simplicity, the *Escherichia coli* expression system is the preferred choice for the production of therapeutic proteins. Generally, the overexpression of eukaryotic proteins in *E coli* is tightly regulated by the inclusion of an inducible promoter [11]. Bacterial systems utilize the *lac* promoter, which is induced by Isopropyl β-D-1-Thiogalactopyranoside (IPTG). In addition, some background expression of host proteins might occur in the bacterial system, even when the *lac* operator sequence is present [12]. The expression of eukaryotic proteins, particularly those of human origin, in such heterologous systems has not been achieved, which is problematic for biophysical or structural biology experiments that require milligram quantities of highly purified protein.

There are several obstacles to the expression of tPA in prokaryotic systems, including disulfide bond formation, rare codon usage, and cytotoxicity [13]. Auto-induction, an alternative to IPTG induction, depends on glucose catabolite repression and lactose (substrate) induction to supply tightly control protein expression [14]. Because of the reduced need for sample processing (e.g., no OD_600_-dependent induction window) and ease by which the culture size can be scaled-up, this system is a very attractive method for achieving high-throughput protein expression. In addition, auto-induction results in a high cell density prior to induction, which often results in several-fold higher yields of the target protein compared with than obtained using conventional IPTG induction [14].

**Figure 1:**
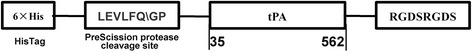
**Recombinant full-length tPA protein.** A scheme showing the functional domains of HisTag-PSP-tPA-RGDS protein (calculated MW 66.7 kDa) generated using auto-induction in *E. co*li Origami 2 cells. The protein was further processed using His-affinity purification and cleavage with PSP to generate a tag-free 528 amino acid full-length tPA with a calculated MW of 60.8 kDa.

To facilitate the structural and functional analysis of tPA, we sought to improve the expression and purification strategies to obtain milligram quantities of purified and untagged full-length tPA protein. tPA with a six-histidine tag (His-tag) was expressed using auto-induction in *E. Coli* (Figure 1). After Ni^2+^-nitrilotriacetic acid (NTA) chromatography and elution with imidazole, the His-tag was cleaved using PreScission Protease (PSP), leaving only two additional residues (Gly and Pro) at the N-terminal. The RGDS, as the integrin binding motif, fused in C-terminal of the recombinant [15]. Gel filtration and fibrin plate experiments suggested that the recombinant tPA was an active monomer in solution.

## Results

### Auto-induction of soluble tPA

The aim of this study was to use bacterially expressed tPA in structural and biophysical experiments that require mg quantities of highly purified protein. To the best of our knowledge, high yields of full-length tPA using these bacterial expression systems have been reported previously only rarely. Therefore, we sought to improve the current expression and purification strategies to obtain milligram amounts of highly purified and untagged tPA protein. BL21 is the most widely used prokaryotic host for protein expression and has the advantage of being deficient in the *lon* and *ompT* proteases. Rosetta host strains are BL21 derivatives designed to enhance the expression of eukaryotic proteins containing rare codons in *E. coli* [16]. Additionally, Origami 2 host strains have mutations in both the thioredoxin reductase (*trx*B) and glutathione reductase (*gor*) genes to enhances disulfide bond formation in the cytoplasm [17]. The expression of tPA was examined in three cell lines using different IPTG concentrations and in the absence of IPTG (data not shown). The cells were then harvested after 4–20 h of induction. All the cell lines showed significant leaky expression in the presence or absence of IPTG (data not shown).

**Figure 2:**
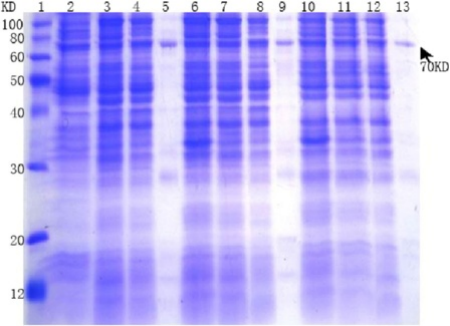
**Soluble expression of tPA in different** ***E. coli*** **strains.** After the auto-induction of tPA expression at 37°C for 24 h. The lysates from *E coli*, supernatant (soluble) fractions, flow-through proteins that did not bind to the column, and recombinant full-length tPA (His-tag-PSP-tPA-RGDS) were analyzed using SDS-PAGE and Coomassie blue staining. Lane 1, protein markers; lane 2, Origami 2 cell lysate; lane 3, Origami 2 supernatant; lane 4, flow-through; lane 5, elution with 200 mM imidazole from Ni^2+^-NTA; lane 6, BL21 lysate; lane 7, BL21 supernatant; lane 8, flow-through; lane 9, elution with 200 mM imidazole from Ni^2+^-NTA; lane 10, Rosetta™ 2 lysate; lane 11, Rosetta™ 2 supernatant; lane 12, flow-through; lane 13, elution with 200 mM imidazole from Ni^2+^-NTA.

A reductive cytoplasmic environment and cytotoxicity are both possible obstacles for the active expression of tPA, a heterologous protein with multiple disulfide bonds, in wild-type *E coli*. Studier developed a reliable protocol for the *lac* operon/promoter-dependent auto-induction of genes in *E coli* [14]. The amount of human proteases expressed using auto-induction is far greater than that achieved using IPTG-based induction. In addition, supplying rare tRNAs (using the Rosetta 2 and Origami 2 strains) did not increase expression compared with BL21 (Figure 2). Origami 2 cells enhanced tPA disulfide bond formation in the cytoplasm; therefore, Origami 2 was the preferred choice for the expression of tPA.

**Figure 3:**
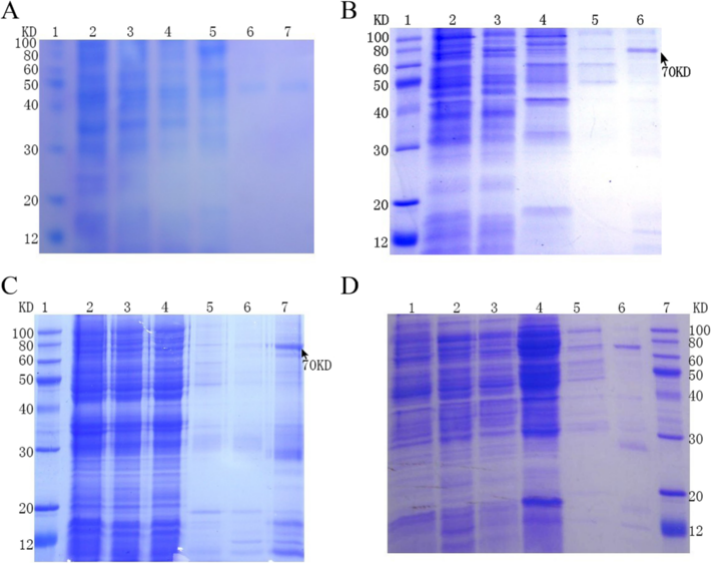
**The auto-induction of Origami 2 (pET28-HisTag-tPA-RGDS) at various temperatures. A,** Recombinant tPA after auto-induction at 25°C. Lane 1, protein marker; lane 2, lysate; lane 3, supernatant; lane 4, flow-through; lanes 5 and 6, washes with 20 and 40 mM imidazole, respectively; lane 7, elution with 200 mM imidazole. **B,** Recombinant tPA after auto-induction at 33°C. Lane 1, protein marker; lane 2, supernatant; lane 3, flow-through; lanes 4 and 5, washes with 20 and 40 mM imidazole, respectively; lane 6, elution with 200 mM imidazole. **C,** Recombinant tPA after auto-induction at 35°C. Lane 1, protein marker; lane 2, lysate; lane 3, supernatant; lane 4, flow-through; lanes 5 and 6, washes with 20 and 40 mM imidazole, respectively; lane 7, elution with 200 mM imidazole. **D,** Recombinant tPA after auto-induction at 37°C. Lane 1, lysate; lane 2, supernatant; lane 3, flow-through; lanes 4 and 5, washes with 20 and 40 mM imidazole, respectively; lane 6, elution with 200 mM imidazole;Lane 7, protein marker.

Next, the effect of temperature was tested to optimize the expression of recombinant tPA. As shown in Figure 3, the recombinant protein was expressed at three different temperatures: 33, 35, and 37°C. Similar yields of recombinant protein were produced at each temperature. These results suggest that expressing the protein at a higher temperature increased the yield of recombinant tPA significantly.

The molecular mechanisms of protein folding have been studied extensively over previous decades both experimental and computationally. However, the refolding of inclusion bodies *in vitro* is a difficult and complex task, particularly for proteins that have multiple domains and complex disulfide bonds. The refolding of tPA, which contains five domains and 17 disulfide bonds, is an even greater challenge. It was presumed that a soluble form of the target protein would be folded in its native state and exhibit biological activity. To this end, different strains harboring the pET28-HisTag-tPA-RGDS plasmid were cultured and auto-induced at 37°C in auto-induction medium, which provides a cell density that is typically several-fold higher than obtained using conventional IPTG induction in LB medium [18]. The auto-induced cells were harvested and lysed by sonication. The cell lysates were separated by high-speed centrifugation, and the expression of tPA in the supernatant and pellets was determined using SDS-PAGE. The Origami 2 strains produced the protein of interest in a soluble form, although ~50% of the tPA was present in the insoluble protein fraction (Figures 2 and 3).

### Purification of soluble tPA protein

To facilitate the purification of tPA, the N-terminal His-tag of pET28-HisTag-tPA-RGDS was captured using metal ion affinity chromatography. This purification strategy prevents the cumbersome refolding processes that lead to extremely poor efficiency, particularly for proteins with several disulfide bonds.

**Figure 4:**
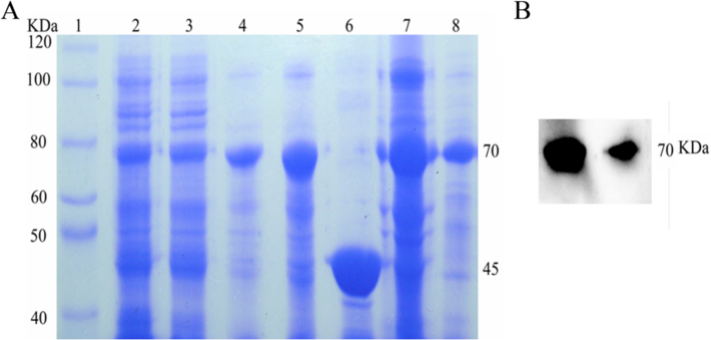
**Purification of recombinant tPA. A,** SDS-PAGE analysis of tPA purity. Lane 1, protein markers; lane 2, lysate; lane 3, supernatant; lane 4, elution with 200 mM imidazole; lane 5, recombinant tPA purified using ion exchange chromatography; lane 6, PSP; lane 7, PSP enzyme reaction mixtures; lane 8, tPA purification using size-exclusion chromatography. **B,** Western blotting of the enzyme reaction products (lane 7 of Figure 4A) and the purified fraction (lane 8 of Figure 4A) using anti-tPA antibodies.

After the cultivation period, cells were harvested by centrifugation, resuspended in cold lysis buffer, and disrupted by sonication. The supernatants containing soluble recombinant tPA protein were applied to an Ni^2+^-NTA affinity column, and were subsequently purified using ion exchange chromatography (DEAE, GE Healthcare, England). The target fractions were then combined and incubated with PSP to cleave the His-tag from tPA. Finally, the recombinant protein was subjected to size-exclusion chromatography. The final purified tPA preparation yielded only one major protein band, at ~65 kDa, after analysis by SDS-PAGE followed by Coomassie Brilliant Blue staining (Figure 4). Although no weak overexpressed band of tPA was observed in total protein lysates from IPTG induction cultures, we used the powerful auto-induction system to generate ~1.8 mg tPA protein (calculated using the BCA method, Thermo Scientific, USA) from an auto-induced *E coli* culture.

### Characterization and activity of the recombinant tPA

SDS-PAGE analysis of the different fractions obtained during purification revealed the progressive enrichment of a ~70 kDa protein (Figure 4). This was the only protein band that was present in the electrophoretically homogeneous final enzyme preparation; it was the was the same size as a tPA monomer (Figure 4). Western blotting confirmed that this band (~70 kDa) was detected by an anti-tPA monoclonal antibody, suggesting that the purified band represents recombinant tPA (Figure 4).

**Figure 5:**
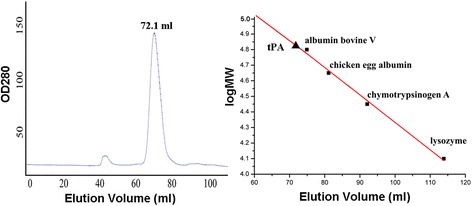
**Size exclusion gel filtration chromatography of tPA using a Superdex 200 column.** tPA (MW ~60.8 kDa) was eluted as a single peak at a volume of 72 ml, which corresponds to a molecular weight of 66.68 kDa on the calibrated column. Standard protein samples (black data points) were used for calibration: albumin bovine V (66.2 kDa), chicken egg albumin (44.2 kDa), chymotrypsinogen A (24.5 kDa), and lysozyme (14.4 kDa).

Previous studies revealed that the P domain of tPA exists as a monomer under native conditions [10]. Therefore, we next aimed to further characterize the molecular weight of tPA in solution. In a size-exclusion chromatography experiment, tPA was eluted in a single peak at 72.1 ml, corresponding to the estimated monomeric mass of ~66.7 kDa (Figure 5). Because tPA has a calculated molecular mass of ~63.8 kDa, the recombinant protease exists mostly as a monomer under native conditions rather than in an intermolecular disulfide-bonded oligomeric form. Both the P domain and full-length tPA exist in a monomeric state in solution, implying that the monomer serves as its Physiological aggregation.

**Figure 6:**
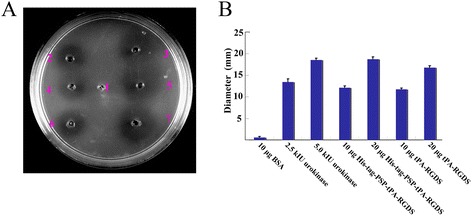
**Lysis on a fibrin plate using urokinase and tPA.** tPA-RGDS; HisTag-PSP-tPA-RGDS; urokinase standard (10 × 10^3^ IU ml^−1^. positive control); BSA (bovine serum albumin standard, negative control). The fibrinolytic activity of tPA was measured and compared with urokinase (positive control) and bovine serum albumin (BSA; negative control). **A,** dot 1, 10 μg BSA; dot 2, 2.5 × 10^3^ IU urokinase; dot 3, 5.0 × 10^3^ IU urokinase (positive control); dot 4, 10 μg His-tag-PSP-tPA-RGDS; dot 5, 20 μg His-tag-PSP-tPA-RGDS; dot 6, 10 μg tPA-RGDS; dot 7, 20 ug tPA-RGDS. **B,** the diameters of the lysis dots on the fibrin plate from at four independent evaluations. The bars indicate standard error of the mean.

Disulfide bonds, which play multiple critical roles in protein stability and function, are often abundantly present in secreted proteins. The functional expression of human proteins with multiple disulfide bonds, such as tPA, in bacterial systems has proved challenging. Therefore, we used fibrin plate assays to assess whether the recombinant tPA was actively folded. Plasminogen was used as the tPA substrate in the medium on the plate. Active tPA can bind to plasminogen and cleave it into plasmin, which degrades fibrin and results a clear lysed zone on the fibrin/agar plate. RGDS, as the binding motif of integrin, will improve the specific affinity of the recombinant tPA to substrate [15]. Similar to tPA, Urokinase could cleave plasminogen into plasmin and was used as a positive control [19]. As is shown in Figure 6A and B, tPA and urokinase exhibited similar halo patterns, suggesting that the His-tag-PSP and RGDS, which were attached to the C- and N-terminus of tPA, respectively, did not affect either tPA receptor-ligand binding or the resulting signal transduction. In addition, the similar diameter of the halo patterns of HisTag-PSP-tPA-RGDS and tPA-RGDS suggests that they exhibit similar amidolytic activities *in vitro*. The activity of recombinant tPA was similar to that of urokinase.

## Discussion

The production of recombinant proteins in their soluble active forms mainly uses eukaryotic and/or prokaryotic expression systems. Because of their low cost and the simplicity of bacterial cultivation, *E. coli* expression systems remain the preferred choice for the production of therapeutic proteins both on a laboratory and an industrial scale. The expression of soluble and active full-length tPA in *E. coli* is a major bottleneck for investigators. Under normal physiological conditions, the reductive environment of *E. coli* cytoplasm makes it challenging to prepare recombinant proteins with multiple disulfide bonds. However, native disulfide bond formation is critical for the proper folding of active proteins. tPA is an important thrombolytic agent that contains five distinct structural domains with 17 disulfide bonds; therefore, it is typically unfolded or misfolded and so inactive when expressed in *E. coli*. Most recent studies have focused on improving the oxidation state of the cytoplasm, for example by selecting the periplasmic space or fusing granules, to express tPA in *E. coli* [20-22]. Nevertheless, because of the rare codon usage, cellular toxicity, and the narrow periplasmic space, the soluble expression of full length tPA has not yet been reported in a prokaryotic system. In the current study, we described a novel and efficient strategy to express and purify high yields of soluble, biologically active tPA.

Although we used the conventional *E. coli* expression system with multiple strategies, such as different host strains, temperatures, and/or IPTG concentrations, we did not observe any overexpressed tPA in the total protein lysates. As a protease, human tPA might be toxic to its host bacteria, which leads to low levels of expression [13]. In addition, the rare code of tPA might hamper the overexpression of large amounts of this protein. Therefore, we explored several novel methods to express soluble tPA. Studier previously developed a reliable protocol for the *lac* operon/promoter-dependent auto-induction of genes in *E. coli* [14]. Auto-induction media contains three different carbon sources: glucose, glycerol, and lactose. At the initial stage, gene expression is suppressed by glucose, and the cells grow to a very high density. Subsequently, the expression is induced in a lactose-dependent manner until the glucose is consumed. Compared with IPTG induction, auto-induction has several advantages including a greater cellular biomass, regulated expression, and reduced sample handling. All three *E. coli* lines (BL21, Rosetta 2, and Origami 2) yielded an obvious target protein band in the cell lysates. The supply of rare tRNAs in Rosetta 2 and Origami 2 did not enhance expression compared with that obtained in BL21 (Figure 2). However, Origami 2 cells enhanced the formation of disulfide bonds; therefore, they were used for the preparation of full-length tPA.

Protein solubility is an important indicator of its correct folding [23,24]. Interestingly, the auto-induction expression system provides advantages for protein expression including enhanced folding and protein solubility. In the current study, ~50% of the tPA was expressed in the soluble form, which represents the first report of soluble tPA expressed using a prokaryotic expression system. Because the recombinant protein is expressed in a soluble form, the purification of tPA could be achieved easily in three steps using affinity systems, ion exchange chromatography, and size-exclusion chromatography (Figure 4). The high proteolytic activity of the recombinant tPA confirmed that an active protease had been expressed successfully (Figure 6). The method used to purify tPA described here is relatively simple and, more importantly, allows the large-scale production of active protein. This suggests that the auto-induction system is an optimal expression system for achieving the high-yield production of soluble tPA in *E. coli.*

Approximately 33% of all known proteases identified to date are serine proteases with a wide variety of functions, including roles in blood clotting, protein digestion, cell signaling, inflammation, and protein processing [25]. As a serine protease, human tPA contains the so-called “classical” catalytic Ser195/His322/Asp371 triad, and cleaves the inactive proenzyme plasminogen to form plasmin, the active enzyme [1,26]. The aggregation state of serine proteases in solution, such as monomers, dimers, and trimers, is related to its biological activity. For some serine proteases, such as thrombin, alpha-synuclein, and Rv3671c from *Mycobacterium tuberculosis*, the monomeric form is active in solution [27-29]. In contrast, some serine proteases such as FAAH amidase, LD carboxypeptidase, and cytomegalovirus protease adopt active homodimers in solution [25]. Herpesvirus proteases, which belong to a unique class of serine proteases that contain a Ser-His-His catalytic triad, exist in monomeric-dimeric equilibrium in solution, and the homodimer is its active form [30,31]. Interestingly, the active form of a mitochondrial serine protease HtrA2 is a pyramid-shaped homotrimer [32].

To characterize the oligomeric state of recombinant tPA in solution, size exclusion chromatography experiments were performed using tPA. Elution peaks were observed at volume of 72.1 ml; this corresponds to an estimated molecular weight of ~66.68 kDa, which is similar to the calculated molecular mass (~60.8 kDa). This suggests that tPA exists mostly as a monomer under native conditions. Importantly, this is the first study to report the aggregation state of full-length tPA. Interestingly, Lee et al. also observed that the P domain of tPA folded correctly as a monomeric protein in solution [10]. Collectively, these results strongly suggest that a monomer of tPA might be required for its proper physiological function.

Recombinant tPA is one of the most promising clinical treatments for improper blood clotting, which might result in a heart attack and stroke [2]. Compared with expression and purification in a eukaryotic system, the scheme described here for full-length tPA is relatively affordable, simple, and, more importantly, is suitable for the large-scale industrial production of active proteins. To our knowledge, this is the first report of the soluble and functional expression of full-length tPA in *E. coli*. The preparation of full-length tPA in its active conformation will allow its three-dimensional structure to be determined and its physiological roles to be characterized.

## Conclusion

In summary, we described a novel method for the soluble expression of large amounts of full-length untagged human tPA in *E. coli*. To the best of our knowledge, this is the first report of the successful large-scale heterologous expression of correctly folded active tPA using a prokaryotic expression system. We used PSP to obtain a highly pure protein preparation with the addition of only Gly and Pro residues at the N-terminus, and a C-terminally fused RGDS to improve the targeting efficiency of tPA in thrombolysis. The typical biophysical properties of tPA, such as its protease activity, were maintained in the purified protein. tPA exists as a monomer in solution, which was verified using size exclusion chromatography, suggesting that the monomer is the structural and active unit of tPA.

## Methods

### Bacterial strains, plasmids, and growth conditions

For recombinant protein expression experiments, chemically competent *E. coli* strains BL21 (DE3), Origami 2 (DE3), and Rosetta™ 2 (DE3) pLysS were transformed using standard protocols (Novagen, USA). Unless otherwise stated, the strains were grown at 37°C in either Luria-Bertani (LB) broth or auto-induction medium with vigorous shaking. They were also grown on tryptone yeast agar [14]. When necessary, 50 μg ml^−1^ kanamycin was added.

### Construction of the fusion expression vector

The gene *tPA* (accession number NM_000930) was amplified from human liver cDNA using the primers tPA-F (5′-AT*GGATCC*ATGCTGGAAGTT***CTGTTCCAGGGGCC***CTCTTACCAAGTGATCTGCAGAGAT-3′; the *Bam*HI site is *italics*, and the PSP site is bold-italics) and tPA-R (5′-AC*AAGCTT*TTA***GCTATCCCCTCGCGAATCCCCTCG***CGGTCGCATGTTGTCACGAATCCA-3′; the *Hind*III site is *italics* and the RGDS peptide is bold-italics). The PSP site enables the proteolytic removal of the His-tag, which can be separated subsequently from the receptor using a Ni^2+^-NTA column (GE Healthcare, Sweden). B*am*HI and *Hin*dIII restriction sites were introduced into the upstream and downstream oligonucleotide primers, respectively (Figure 1). PCR was performed using *Pfu* DNA polymerase (Promega, USA) with the following conditions: denaturation at 94°C for 5 min, followed by 25 cycles of 94°C for 40 s, 58°C for 40 s, and 72°C for 180 s, with a final elongation step at 72°C for 10 min. Two blanks that contained all the reaction components except the primers or cDNA, respectively, were used as controls. The PCR fragments were double-digested with *Bam*HI and *Hind*III, and then subcloned into pET28a expression vector (Novagen, USA) that had been pre-digested with the same enzymes. The vector contained an N-terminal His-tag and a C-terminal RGDS sequence. The presence of the insert in the recombinant plasmid was verified using DNA sequencing. The resulting plasmid for the expression of tPA in *E. coli* was named pET28-HisTag-tPA-RGDS.

### Soluble expression and purification of tPA

The *E coli* strains BL21 (DE3), Origami 2 (DE3) and Rosetta™ 2 (DE3) pLysS were transformed with the recombinant pET28-HisTag-tPA-RGDS plasmid. The recombinant protein was then expressed using auto-induction at 37°C overnight, as described previously [14]. The transformed cells were selected on LB medium plates supplemented with 50 μg ml^−1^ kanamycin. For pre-cultures, 5 ml of LB medium was inoculated using a single colony picked directly from agar plates, and then cultivated at 37°C with shaking at 180 rpm for 12 h. For the main cultivation, five separate 200 ml cultures of auto-induction media containing kanamycin (50 μg ml^−1^) were inoculated with 2 ml of the pre-culture in flasks shaking at 250 rpm for 15 h at 37°C. For temperature optimization, the bacterial cultures were grown at four different temperatures (25, 33, 35, and 37°C) and auto-induced for 30, 24, 20, and 15 h, respectively.

At the end of the cultivation period, cells were harvested by centrifugation at 4000 rpm for 10 min. The bacterial pellets were resuspended in 100 ml cold lysis buffer (20 mM Tris pH 8.0 and 300 mM NaCl), and disrupted by sonication (20 min at 30% amplitude, with 3 s on and 6 sec off cycles) in an ice-bath to avoid overheating the samples. All purification steps were performed at 4°C. The cell debris was removed by centrifugation at 15 000 rpm for 30 min at 4°C. The supernatants containing soluble recombinant tPA protein were applied to Ni^2+^-NTA affinity resin (Qiagen, Germany) that had been equilibrated with lysis buffer. Non-specifically bound proteins were eluted using 200 ml wash buffer (20 mM Tris pH 8.0, 300 mM NaCl, and 30–50 mM imidazole), and the target protein was eluted in ~60 ml elution buffer (lysis buffer containing a constant concentration of 300 mM imidazole). The eluted protein was then purified further using ion exchange chromatography (DEAE, GE Healthcare) and separated using a linear gradient elution of NaCl (0–500 mM with 20 mM Tris–HCl pH 8.0). Then, fractions containing the protein were combined and incubated with PSP (made in our laboratory) overnight at 4°C to cleave the His-tag from tPA. The reaction products were centrifuged, and the supernatant was separated using size-exclusion chromatography (Superdex 200, GE Healthcare) with a mixture of 200 mM NaCl and 20 mM Tris–HCl pH 8.0. Finally, the fractions containing tPA were combined, concentrated, and buffer-exchanged into the final buffer (5 mM Tris–HCl pH 8.0 and 100 mM NaCl) using a 5-kDa cutoff Millipore Amicon concentrator. They were then stored at 193 K for subsequent studies. The concentration of the final purified recombinant protein was ~1.0 mg ml^−1^.

### SDS electrophoresis

To check the purity of the tPA preparations, SDS-PAGE was performed using a 5% stacking gel and a 10% separating gel in Tris-glycine running buffer. Unless otherwise stated, samples were diluted to a protein concentration of 0.1–0.5 mg ml^−1^ before loading. Samples were boiled 1:2 with loading buffer (70 mM SDS, 100 mM dithiothreitol [DTT], 10% [v/v] glycerol, 0.05 M Tris pH 6.8, 0.06% [w/v] pyronin G). Gels were run in a vertical Mini-PROTEAN Tetra MP4 apparatus (Bio-Rad, USA) and stained with Coomassie Brilliant G250.

### Western blotting

Proteins were separated using SDS-PAGE in Tris-glycine buffer, and transferred to polyvinylidene difluoride (PVDF) membranes overnight using a semi-dry electroblotting apparatus (Bio-Rad). The membranes were washed with TBST (10 mM Tris–HCl pH 8.0, 150 mM NaCl, and 0.05% Tween 20) and blocked with 3% (w/v) bovine serum albumin (BSA) for 1 h at room temperature. Subsequently, the membranes were incubated overnight with goat-anti-tPA antibodies (sc-5239, Santa Cruz, CA, USA) at 4°C with permanent shaking, and were then washed and incubated with horseradish peroxidase (HRP)-conjugated secondary antibodies. Finally, the bound antibodies were detected using enhanced chemiluminescence (Millipore, Billerica, MA, USA).

### Oligomeric state determination

Size exclusion chromatography was performed using a Superdex 200 column (GE Healthcare) as described previously [33]. The protein of interest or molecular mass standards were applied to the Superdex 200 (GE Healthcare) column, and eluted using 50 mM Tris–HCl pH 8.0 and 150 mM NaCl. The protein molecular weight standards used were albumin bovine V (66.2 kDa), chicken egg albumin (44.2 kDa), chymotrypsinogen A (24.5 kDa), and lysozyme (14.4 kDa). The peak elution volumes (measured at 280 nm) were used to calculate the standard curve using the equation log MW = −0.01712 Ve + 6.05667, with R^2^ = 0.9726. tPA was diluted to 1 mg ml^−1^ in buffer (50 mM Tris–HCl pH 8.0 and 150 mM NaCl), and then loaded on the column. tPA was eluted as a single peak in a volume of 72.1 ml, corresponding to an estimated molecular weight of 66.68 kDa.

### Assay to assess the activity of the recombinant protein

The plasminogen activation activity of tPA was assessed using agarose-fibrin plates according to methods described previously with minor modifications [34-36]. The agarose-fibrin plates were prepared as follows: 1.0 IU thrombin, 0.75 g plasminogen, and 30 IU human fibrinogen were added to 15 ml of 1.0% agarose gel that had been dissolved in normal saline at 45–55°C. The mixture was incubated at room temperature for 30 min. The sample was then loaded onto the plate, which was incubated at 37°C for 24 h. To determine the activity of the recombinant tPA, the standard (urokinase) was serially diluted and spotted onto the fibrin plates. The activity of the recombinant tPA was then measured according to the diameter of the clear zone on the fibrin plates.
